# Determinants of bone mass and bone size in a large cohort of physically active young adult men

**DOI:** 10.1186/1743-7075-3-14

**Published:** 2006-02-15

**Authors:** JA Ruffing, F Cosman, M Zion, Susan Tendy, P Garrett, R Lindsay, JW Nieves

**Affiliations:** 1 Clinical Research and Regional Bone Centers, Helen Hayes Hospital, West Haverstraw, New York, USA; 2 Departments of Medicine and Epidemiology, College of Physicians and Surgeons of Columbia University, New York, USA; 3 United States Military Academy, West Point, New York, USA

## Abstract

The determinants of bone mineral density (BMD) at multiple sites were examined in a fit college population. Subjects were 755 males (mean age = 18.7 years) entering the United States Military Academy. A questionnaire assessed exercise frequency and milk, caffeine, and alcohol consumption and tobacco use. Academy staff measured height, weight, and fitness. Calcaneal BMD was measured by peripheral dual-energy x-ray absorptiometry (pDXA). Peripheral-quantitative computed tomography (pQCT) was used to measure tibial mineral content, circumference and cortical thickness. Spine and hip BMD were measured by DXA in a subset (n = 159). Mean BMD at all sites was approximately one standard deviation above young normal (*p *< 0.05). African Americans had significantly higher hip, spine and heel BMD and greater tibial mineral content and cortical thickness than Caucasians and Asians. In Caucasians (n = 653), weight was a significant determinant of BMD at every skeletal site. Prior exercise levels and milk intake positively related to bone density and size, while caffeine had a negative impact. There was an apparent interaction between milk and exercise in BMD at the heel, spine, hip and tibial mineral content and cortical thickness. Our data confirm the importance of race, body size, milk intake and duration of weekly exercise as determinants of BMD and bone size.

## Introduction

The Surgeon General recently highlighted bone health as an important public health issue in the United States and 20% of osteoporosis occurs in men. [1,2] Bone mass accumulates throughout childhood and adolescence until peak bone mass is reached during the third decade of life. When higher BMD is attained at a young age (higher peak bone mass) there is a subsequent reduction in the risk of childhood fractures, stress fractures, and possibly osteoporosis and related fractures later in life. [3-9] Studies indicate that a larger bone size is also related to a reduced risk of fracture. [10]. Genetic factors account for between 60–80% of the variance in peak bone mass and bone size. [11-14] However, an individual male may not achieve his genetically determined bone mass/size, if environmental and lifestyle conditions are not permissive

High levels of physical activity and adequate calcium intake have been shown to improve accrual of peak bone mass, although data in males are limited. [15-17]. Numerous studies indicate that tobacco use and excessive alcohol and caffeine consumption are associated with lower bone mass in young adults [18-24], however, there is little known about the impact of these lifestyle factors on bone size. The purpose of this cross sectional study was to examine the influence of milk intake and exercise levels in the prior year, body size and race on bone mineral density and bone size in a physically fit male college age population.

## Methods

### Subjects

Subjects were college-aged males recruited from the United States Military Academy Class of 2002, West Point, NY. The Institutional Review Board (IRB) of Keller Army Community Hospital (KACH), West Point, NY, approved the study. During their first week at the Academy, all members of the class attended a presentation describing the study objectives and associated risks, and were then invited to participate. The military academy has stringent medical requirements for attendance; therefore, no exclusion criteria were required for this study. Approximately 70% of all the males in the class (n = 755) provided written informed consent.

### Lifestyle assessments

A self-administered baseline questionnaire was used to assess exercise, lifestyle and dietary habits in the year prior to Academy entrance. Race data was provided from Academy records. Exercise (weekly average) was categorized as: 1–3, 4–6, 7–10, or 11 + hours /week during the prior year. Daily milk consumption was assessed as: <1, 1–2, or 3 or more 8 ounce glasses a day. Four questions assessed calcium intake including daily milk consumption and weekly servings of yogurt, cheese and high calcium content vegetables (0, 1–3, 4–6 or 7 + serving per week). Daily caffeine consumption was divided into three categories: none, 1 to 3 and >3 caffeine containing drinks a day. Alcohol intake was assessed using 5 categories: less than once a month, 1–3 times a month, 1 to 2 times a week, and 3 to 5 times a week or daily. Tobacco use was assessed by type (dip, chew, or cigarettes), dose and duration. Cadets were given a baseline fitness test. The test had three components: a 2 mile run, 2 minutes of push ups and 2 minutes of sit ups. All three events are graded from 0–100 with better performers receiving a higher score.

### Bone densitometry and anthropometric measures

Academy personnel measured each cadet's height and weight in their physical fitness uniform that consists of a standard issue t-shirt, nylon shorts and socks. These measurements were used to calculate Body Mass Index (BMI: weight (kg)/height (m^2^)).

BMD (g/cm^2^) of the left calcaneus was measured by DXA (pDXA; Pixi, Lunar, Madison, WI) in all cadets. Peripheral quantitative computed tomography (pQCT; Stratec, Germany XCT-2000) was used to image a single slice at the two-third distal tibia. The distal third of the tibia was determined by a manual measurement of tibial length between the base of the patella and the styloid process to the closest centimeter. The 2/3 site was then located by the pQCT scanner after placing a positioning light of the gantry above the styloid process. Bone mineral content (mg per 1 mm slice of bone), bone density (mg/cm^3^), cortical thickness (mm) and periosteal circumference (mm) were measured. Cortical thickness was derived using the circular ring model, using a threshold of 710 mg/cm^3 ^to define cortical bone. This model calculates a mean cortical thickness from measures of total bone area and cortical bone area. In a randomly selected subset (n= 159) of male cadets, total hip and spine BMD (lumbar vertebral bodies: L2–L4) were measured using standardized positioning devices and the high-resolution software mode in a mobile DXA scanner (DPX-IQ, Lunar, Madison WI). The short-term coefficient of variation for each bone measurement was calculated by scanning 10 individuals on each machine twice. The coefficient of variation for bone density in vivo was 1.0%, 1.2%, 1.5% and 1.5% for the calcaneus by pDXA, tibia by pQCT, and spine and total hip by DXA respectively.

### Statistical analysis

The relationships between lifestyle factors measured continuously (e.g. height and weight) and bone variables were examined using correlation analyses and, where appropriate, with linear regression to control for potential confounders. Effects of categorical lifestyle variables such as alcohol consumption were coded from 1 to n based on the number of categories assessed. Differences in the effect of categorical variables on bone indices were assessed using analysis of variance using Sidak post hoc analysis. The relationships between BMD at different skeletal sites with fitness measures including running score were assessed by linear regression. Comparisons between cadets and reference populations were performed using *t*-tests for independent groups. For each skeletal site, a step wise multiple regression model was created for Caucasian males evaluating all covariates that had biologic plausibility and were significant in the univariate analysis. Creating a cross-product term and evaluating it as an independent predictor in the site-specific regression models evaluated the interaction of milk intake and physical activity. The level of significance for alpha was set at 0.05 for all statistical tests. All analyses were performed with SPSS statistical software (Version 13.0 for Windows, SPSS Inc., Chicago IL.)

## Results

### Population characteristics

There were 755 male cadets enrolled in the study of which eighty six percent were Caucasian (n = 653), eight percent were African American (n = 64) and six percent were Asian (n = 38). The mean (± SD) age at entry was 18.7 (± 1.03) years. Table 1 provides a summary of the distribution of lifestyle variables separately for Caucasians, African Americans and Asians. Most cadets (74%) exercised more than 7 hours per week and had an average calcium intake over 1000 mg /day, with the majority of calcium (64%) coming from milk. Only 5.4% of male cadets smoked and only 18% consumed alcohol more than once per week. Caucasians consumed significantly more alcohol than either Asians or African Americans (*p *< 0.02) while Asians consumed significantly less caffeine than any other race (*p *< 0.01) There were no significant differences in the lifestyle characteristics between those men that had central DXA measurements and the whole group; however, cadets with central DXA had a significantly shorter height (1.03 inches) and lower weight (10.03 pounds) than the entire group of men.

**Table 1:** Lifestyle variables for the year preceding academy entrance

**Variable**	**Mean ± SD (Caucasians)**	**Mean ± SD (African Americans)**	**Mean ± SD (Asians)**
Age (years)	18.7 (± 1.05)	18.5 (± 0.94)	18.5 (± 0.73)

**Variable**	**Percentage (n)**	**Percentage (n)**	**Percentage (n)**

Exercise (hrs/week)			
1–3	5.5 (36)	6.3 (4)	10.5 (4)
4–6	19.5 (127)	23.3 (15)	19.5 (6)
7–10	30.9 (201)	28.1 (18)	30.9 (13)
11+	44.1 (287)	42.2 (27)	44.1(15)
Milk (glass/day)			
0	4.4 (29)	4.7 (3)	7.9 (3)
< 1	17.0 (111)	28.1 (18)	13.2 (5)
1–2	47.4 (309)	43.8 (28)	50.0 (19)
3+	31.1 (203)	23.4 (15)	28.9 (11)
Caffeinated (Drinks/day)			
None	17.1 (111)	14.3 (9)	36.8 (14)
1–3	70.3 (457)	76.2 (48)	57.8 (22)
3+	12.6 (82)	9.5 (6)	5.3 (2)
Alcohol Consumption			
Less than once a month	57.8 (375)	78.1 (50)	66.7 (22)
1–3 times a month	22.7 (147)	15.6 (10)	30.3 (10)
1–2 times a week	14.6 (95)	4.7 (3)	9.1 (3)
3–5 times a week	4.0(26)	1.6(1)	0.0 (0)
daily	0.9 (6)	0.0 (0)	0.0 (0)
Smokers	5.1 (41)	4.7 (3)	13.1 (5)

### Racial differences

There were significant racial differences in height, weight, and BMI as shown in Table 2. Asian males weighed less, were shorter and had a lower BMI than Caucasians or African Americans (all *p *< 0.013). Mean calcaneal BMD in African-Americans was significantly higher than either Caucasian or Asian males and, in all races, was higher than the manufacturer's population average in all races (Table 2; *p *< 0.03) [25]. African Americans had significantly higher tibial mineral content (*p *< 0.03). Tibial geometry also differed by race; African-Americans had significantly larger periosteal circumference and greater cortical thickness than Asians or Caucasians. African Americans also had significantly higher BMD of the hip (*p *< 0.008) than either Caucasians or Asians but there were no *significant *differences in spine BMD after controlling for weight. Racial differences have been well reported. [26,27]

**Table 2:** Racial differences in anthropometric measures and bone mineral density (mean ± SD)

**Anthropometric Variables**	**Caucasians n = 653**	**African Americans n = 64**	**Asians n = 38**
Height (inches)	70.2 (± 2.7)	69.5(± 2.9)	65.6(± 2.4)^a^
Weight (pounds)	173.4 (± 29.0)	172.5(± 29.4)	154.8(± 22.3)^a^
BMI (kg/m^2^)	24.7 (± 3.4)	25.1(± 3.6)	23.1(± 2.9)^a^
**Bone Mineral Density/Size**			
Calcaneal BMD (g/cm^2^)	0.706 (± 0.13)	0.852 (± 0.17)^a^	0.666 (± 0.14)
Lumbar Spine BMD (g/cm^2^)	1.282 (± 0.13)	1.309 (± 0.19)	1.272 (± 0.13)
Total Hip BMD (g/cm^2^)	1.244 (± 0.15)	1.356 (± 0.20)^a^	1.159 (± 0.09)
Distal Tibia BMD (mg/cm3)	810 (± 85.1)	813. (± 84.8)	811 (± 92.3)
Tibial Mineral Content (mg)	354.8 (± 44.7)	390.0 (± 53.9)^a^	344.6 (± 40.3)
Cortical Thickness (mm)	6.17 (± 0.78)	6.41 (± 0.90)	6.06 (± 0.79)
Periosteal Circumference (mm)	73.7 (± 5.7)	76.3^a ^(± 5.8)^a^	72.9 (± 5.1)

^a ^p < 0.05 vs. other races

### Predictors of bone density in Caucasian males

#### Calcaneus

**Figure 1:**
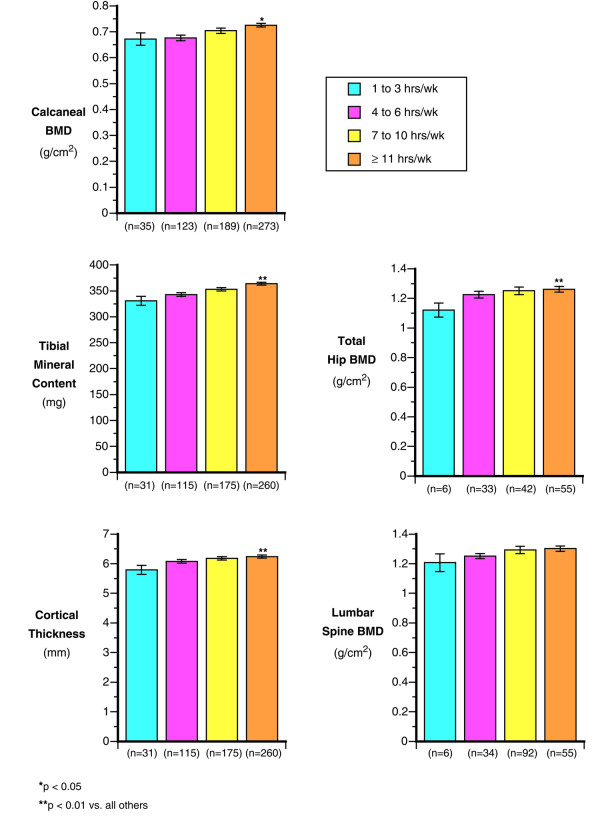
The impact of weekly average exercise in the prior year (shown as: 1–3, 4–6, 7–10, or 11 + hours/week) on various bone variables.

Height and weight were correlated with calcaneal BMD (r = 0.33, r = 0.53, respectively, both *p *< 0.001). For each additional ten pounds of weight there was a 4% increase in heel BMD. Prior exercise history was positively related to calcaneal BMD with approximately 3% higher calcaneal BMD with each 3 additional hours of exercise per week. (*p *< 0.002 for trend; Figure 1).

**Figure 2:**
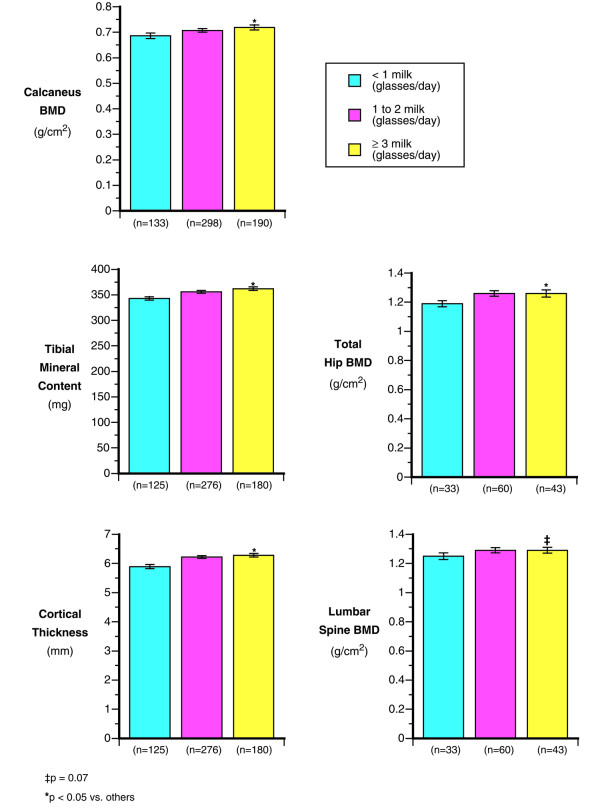
Impact of milk intake in the prior year (shown as: <1, 1–2, or 3 or more 8 ounce glasses a day) on various bone variables.

Caffeine consumption was negatively related to calcaneal BMD in Caucasians (2.5% lower for 2 or more cups per day consumed). Lower milk consumption translated into a 4% lower calcaneal BMD between cadets with low (1 or fewer glasses) vs. higher daily milk intake (Figure 2; *p *< 0.03). Total calcium was related to heel BMD (r = 0.12; *p *< 0.01), but daily dairy calcium had a higher correlation (r = 0.17 ; *p *< 0.01). Alcohol and tobacco intake were not related to calcaneal BMD (both *p *< 0.5). Table 3 presents the results of a stepwise regression analysis, with the best predictors of calcaneal BMD being height, weight, caffeine intake and the cross product of milk and exercise.

**Table 3:** Skeletal site regression models

Calcaneal Density	0.026 +	0.004(height) +	0.002(weight) +	0.003(milk*exercise) -	0.019(caffeine)	
(SE)	(0.117)	(0.002)	(0.001)	(0.001)	(0.008)	
*p*	(0.826)	(0.02)	(0.001)	(0.006)	(0.02)	
*R*^2 ^= *0.34*						
Lumbar Spine Density	0.810 +	0.003(weight) -	0.114(smoke) +	0.414(milk*exercise) -	0.095(milk) -	0.098(exercise)
(SE)	(0.180)	(0.001)	(0.051)	(0.014)	(0.043)	(0.044)
*p*	(0.001)	(0.004)	(0.001)	(0.017)	(0.029)	(0.027)
*R*^2 ^= *0.35*						

Total Hip Density	1.608 +	0.003(weight) -	0.012 (height) -	0.130 (smoke) +	0.006(milk*exercise)	
(SE)	(0.324)	(0.001)	(0.005)	(0.059)	(0.003)	
*p*	(0.001)	(0.004)	(0.001)	(0.017)	(0.049)	
*R*^2 ^= *0.26*						

Tibial Density	1328.999 -	9. 147(height) +	1.791(milk*exercise) +	0.633(run score)	+ 0.349(weight)	
(SE)	(105.744)	(1.628)	(0.930)	(0.224)	(0.169)	
*p*	(0.001)	(0.001)	(0.055)	(0.005)	(0.04)	
*R*^2 ^= *0.08*						

Tibial Mineral Content	127.523 +	1.099(weight) +	0.515(run score) +	0.006(milk*exercise) -	7.588(caffeine)	
(SE)	(15.774)	(0.060)	(0.94)	(0.395)	(2.838)	
*p*	(0.001)	(0.001)	(0.001)	(0.001)	(0.008)	
*R*^2 ^= *0.45*						

Cortical Thickness	7.192 +	0.008(height) +	0.38(milk*exercise) -	0.039(height)		
(SE)	(0.870)	(0.008)	(0.042)	(0.014)		
*p*	(0.001)	(0.001)	(0.001)	(0.005)		
*R*^2 ^= *0.11*						

Periosteal Circumference	27.85 +	0.081(weight) +	0.475(height) -	0.768(caffeine)		
(SE)	(5.59)	(0.008)	(0.088)	(0.376)		
*p*	(0.001)	(0.001)	(0.001)	(0.041)		
*R*^2 ^= *0.31*						

#### Tibial mineral content

Weight and height were significantly correlated to tibial mineral content (r = 0.60 and r = 0.39 respectively: *p *< 0.001). Prior exercise history was positively correlated to tibial mineral content (r = 0.23; *p *< 0.001) and there was a significant 5% difference in tibial mineral content between those in the highest exercise group versus all others (*p *< 0.001; Figure 1). Milk consumption was related to tibial mineral content (Figure 2) and there was a 5% lower tibial content between those consuming less than 1 glass of milk per day as compared to those consuming more milk (*p *< 0.03). Total calcium intake was correlated to tibial mineral content (r = 0.16; *p *< 0.10), Alcohol and tobacco use had no apparent relationship with tibial mineral content (both *p *< 0.2). Higher caffeine intake was related to a significant decrease in tibial mineral content (*p *< 0.05).

The regression model that best predicted mineral content in the tibia included weight, run score, caffeine, and the cross product term of milk and exercise (Table 3).

#### Tibial size

Weight was correlated with cortical thickness (r = 0.24; *p *< 0.001) and periosteal circumferences (r = 0.52; *p *< 0.001). While height was correlated with periosteal circumference (r = 0.43; *p *< 0.001). Prior exercise significantly correlated to cortical thickness (r = 0.13; *p *< 0.002) and periosteal circumference (r = 0.18; *p *< 0.005). Cadets in the highest exercise group had 5.8% higher cortical thickness compared to those in the lowest exercise group (*p *< 0.04; Figure 1).

**Figure 3:**
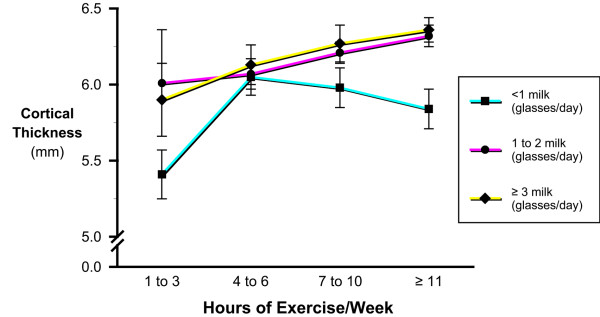
Interaction between milk and exercise during the prior year on cortical thickness of the tibia.

Alcohol intake had no apparent influence on tibial size (periosteal circumference; *p *< 0.97) or cortical thickness (*p *< 0.24). Total calcium intake was positively correlated to cortical thickness (r = 0.18 ;*p *< 0.001) and perisoteal circumference (r = 0.10; *p *< 0.001). Daily milk consumption was positively associated with cortical thickness and periosteal circumference, such that cortical thickness was significantly lower (5.9%) in the lowest milk intake categories compared to the highest milk intake group (*p *< 0.04; *p *< 0.01; Figure 2). There was a significant interaction between milk intake and prior exercise in relation to cortical thickness (Figure 3). Males with high prior exercise duration only showed a skeletal benefit of the exercise if milk intake was greater than one glass per day.

The models best predicting cortical thickness and periosteal circumference are provided in Table 3.

#### Lumbar spine and total hip

Weight was significantly correlated with both hip and spine BMD (r = 0.41 and r = 0.52 both *p *< 0.001). Each additional 10 pounds of weight was associated with a 0.03 g/cm^2 ^(2.4%) incremental gain of BMD of the spine and 0.05 g/cm^2 ^in the hip (4 %).

Those cadets who had the highest prior exercise levels (>11 hours per week) had 5.1% higher hip BMD (*p *< 0.016) and 5.4% higher spine BMD (*p *< 0.008) then those with lower levels (Figure 1). Total calcium was correlated with the hip (r = 0.20; *p *< 0.02) but not the spine. Caffeine and alcohol intake had had no apparent effect on hip BMD (both *p *< 0.15). Cadets with low milk intake (<1 glass/day) had 4% lower spine and 6% lower hip BMD compared to those cadets whose intake of milk was 3 or more glasses per day *p *< 0.07 and *p *< 0.04 respectively). However, smoking was negatively associated with both the hip (*p *< 0.03) and spine (*p *< 0.04). In smokers, spine and hip BMD measured an average of 8–10% lower than in non-smokers (both *p *< 0.04).

The most parsimonious regression model for spine BMD included weight, milk consumption, past exercise, smoking and the product term of milk and exercise. The addition of height to the same factors in the hip model (Table 3) explained 35% of the variance in hip BMD.

## Discussion

In this large sample of fit males, healthy lifestyles, including exercise and milk consumption, are critical for optimal bone density and bone size. There was also an apparent synergistic effect between milk consumption and exercise with regard to bone density at multiple skeletal sites as well as on the cortical thickness of the tibia. It was also found that smoking and excessive caffeine consumption could have a negative impact on the skeleton in this cohort.

Racial differences in bone mass, bone geometry and size still exist even when controlling for height and weight, although as shown in other studies, [28,29] the magnitude of those differences is smaller after controlling for body size. The differences in tibial cortical thickness and circumference have not been shown previously and may help explain the lower fracture rates in African Americans. Our study confirmed the findings of other studies that racial differences in BMD exist, [27,30] and there are also differences in bone size and geometry even after controlling for height and weight. Limited power precluded us from have race specific models for each skeletal site.

Weight and height were consistent predictors of bone density at all sites as has been shown in prior studies; however weight and height are also related to tibial size. This is not surprising since genetic factors, including height and weight, can account for between 60–80% of the variation in BMD. [13,14,31-33] In this study, significant racial differences in BMD at the total hip and tibial mineral content persisted even after controlling for weight.

The positive effect of exercise on BMD is supported by this study. [34-36] Even in a fit population, with BMD at the upper end of the normal range, higher levels of exercise were associated with higher bone mass. Periosteal circumference, or size of the tibia, as well as the tibial mineral content were significantly influenced by prior exercise levels presumably due to the impact of increased mechanical loading from exercise. Since there may be few periods in life where bone size can be altered, taking advantage of this relationship in young adults may be important to a future reduction in fracture risk.

In this cohort of males, milk was significantly related to cortical thickness, hip and heel BMD and tibial mineral content. These findings support prior studies suggesting that milk intake is an important factor in increasing bone density and bone size in young adults. [37,38] Milk intake may be a marker for calcium intake alone or for an overall healthy diet, or may reflect the influence of some other important nutrient in milk such as protein or vitamin D. It is likely that the skeletal benefit of milk based calcium is a result of many factors. Although there was not a significant interaction, the negative effect of caffeine was minimized at higher levels of milk intake, as has been previously reported. [39] This may indicate that the negative effect of caffeine on bone may be due primarily to reduced milk intake or to a reduced absorption of calcium at higher levels of caffeine intake. The negative dose response relationship between caffeine and calcaneal BMD has been reported in other populations. [40,41]

Smoking also had a deleterious effect on hip and spine BMD in these otherwise healthy young men, as has been previously reported. [18,20,40,42] This may relate to reduced osteoblast function in males who smoke [43] or perhaps a more rapid metabolism of hormones (estrogen and testosterone) that may affect BMD in men. [44]

A unique aspect of our study was the ability to determine factors that predict tibial size and mineral content in these young men. A skeleton that is more resistant to fractures should not only have a higher mineral content or density but also a larger bone size (circumference) and cortical thickness. The critical periods during which bone size can be maximized are likely to occur at an early age, prior to attainment of peak bone mass and this could be evaluated in this young adult cohort. Tibial size (periosteal circumference) was found to be related to genetically modulated characteristics including height and weight, as well as lifestyle factors including exercise and nutrition.

Mathematical models, including linear regression, are limited to evaluating the determinants of bone health in an additive way. This may not represent the true biologic relationship. Therefore we examined a number of cross product terms. The milk -exercise cross product proved to be significant in the tibial mineral content and cortical thickness model as well as for BMD of the calcaneus, hip, and spine. The milk – exercise interaction has been reported elsewhere and is biologically plausible. [45-47] The mechanical stimulus created by the exercise forces the bone to remodel to adapt to these new loads and repair damage incurred by fatigue created by the strains of exercise. The higher milk consumption creates a greater supply of calcium and possibly other needed nutrients and therefore, a larger more dense bone. The significant cross product term of milk and exercise is indicative of a synergistic interaction between the daily milk consumption and regular exercise.

This study has a number of limitations. First, it is not possible, as with all cross sectional studies, to establish a temporal relationship between diet, exercise and the acquisition of greater bone mass. Second, our baseline questionnaire was self-administered and therefore is limited by individual recall. The dietary questionnaire was limited because of time constraints and did not allow an assessment of factors that may be important including vitamin D, protein, other nutrients and total caloric intake. In addition we only assessed exercise history and milk consumption in the past year and we cannot know whether these behaviors were consistent over time. An additional limitation of the study may be its external validity: our study population is clearly more fit than the normal college age population. Therefore these findings may not be readily generalizable, although this study indicates that lifestyle variables, including exercise, may still have an impact on BMD. Finally, the statistical power, calculated post hoc ranged from 57 to 92 %. Some of the risk factors including smoking and alcohol intake could not be fully explored because of relatively small sample sizes, limited variability and limited power.

In conclusion, BMD and bone size are determined by a complex combination of genetic, lifestyle, and nutritional factors. In our study height, weight, prior exercise and milk intake, smoking and caffeine intake were the most frequent and important predictors of bone mineral density and bone size in these fit young men. In addition the interaction between milk consumption and exercise history was a significant predictor of bone size at the tibia and BMD at all skeletal sites. This information can assist in confirming intervention strategies for parents, schools and pediatricians during the critical years of bone development and the attainment of peak bone mass as suggested by the Surgeon General's report on Bone Health [1]. These bone healthy behaviors should include higher milk intakes, adequate levels of exercise, limited caffeine intake and avoidance of tobacco products. Promotion of these behaviors associated with higher peak bone mass in males may help prevent stress fractures and osteoporosis and related fractures later in life.
